# Multiple Physiological Signals Fusion Techniques for Improving Heartbeat Detection: A Review

**DOI:** 10.3390/s19214708

**Published:** 2019-10-29

**Authors:** Javier Tejedor, Constantino A. García, David G. Márquez, Rafael Raya, Abraham Otero

**Affiliations:** Department of Information Technology, Escuela Politécnica Superior, Universidad San Pablo-CEU, CEU Universities, Campus Montepríncipe, Boadilla del Monte, 28668 Madrid, Spain; constantinoantoniogm@gmail.com (C.A.G.); david.gonzalez.marquez@gmail.com (D.G.M.); rafael.rayalopez@ceu.es (R.R.); abraham.otero@gmail.com (A.O.)

**Keywords:** fusion, electrocardiogram, physiological signals, heartbeat detection

## Abstract

This paper presents a review of the techniques found in the literature that aim to achieve a robust heartbeat detection from fusing multi-modal physiological signals (e.g., electrocardiogram (ECG), blood pressure (BP), artificial blood pressure (ABP), stroke volume (SV), photoplethysmogram (PPG), electroencephalogram (EEG), electromyogram (EMG), and electrooculogram (EOG), among others). Techniques typically employ ECG, BP, and ABP, of which usage has been shown to obtain the best performance under challenging conditions. SV, PPG, EMG, EEG, and EOG signals can help increase performance when included within the fusion. Filtering, signal normalization, and resampling are common preprocessing steps. Delay correction between the heartbeats obtained over some of the physiological signals must also be considered, and signal-quality assessment to retain the *best* signal/s must be considered as well. Fusion is usually accomplished by exploiting regularities in the RR intervals; by selecting the most promising signal for the detection at every moment; by a voting process; or by performing simultaneous detection and fusion using Bayesian techniques, hidden Markov models, or neural networks. Based on the results of the review, guidelines to facilitate future comparison of the performance of the different proposals are given and promising future lines of research are pointed out.

## 1. Introduction

Cardiovascular diseases (CVDs) are the leading cause of death in the world, and they are projected to remain so [1]. However, at least 80% of heart-disease deaths could be avoided [1]. Therefore, assessing the cardiovascular states of patients has become an important concern in all health-care systems. The diagnose of CVDs usually starts with heartbeat detection on the electrocardiogram (ECG). R-peak detection from single-lead ECGs has been extensively studied [2,3,4,5,6,7,8,9,10,11,12,13]. Although it may be considered an easy task under ideal conditions (proper sensor location on the patient’s body, good contact of the electrodes with the skin, bedridden and completely still patient, and absence of electrical noise sources, among others), these conditions are usually not maintained throughout the entire recording in real applications [14]. ECG recordings usually contain noise, baseline wander, and artifacts caused by patient movement or even sensor disconnections, which may result in intervals with total signal loss. Multi-lead ECG approaches for heartbeat detection have also been presented [15,16,17,18,19,20,21], though these also tend to fail with ECG data corrupted with high levels of noise; ECG noise (especially caused by patient movements and electrical interference) tends to be present in all the available leads.

Technological advances have led to more capable monitoring systems, which usually permit the synchronous recording of multiple physiological signals, including the ECG (single lead or multi-lead), continuous blood pressure (BP), arterial blood pressure (ABP), respiration (RESP), photoplethysmogram (PPG), electroencephalogram (EEG), electrooculogram (EOG), and electromyogram (EMG), among others. This is especially relevant in Intensive Care Units (ICUs), where different vital parameters of the patient must be monitored. This multi-modal scenario provides an opportunity for improving the accuracy of ECG-based heartbeat detectors, since many of the monitored signals are related to or influenced by cardiovascular activity. The redundant information extracted from them can be used to support the heartbeat detection when the ECG signal is noisy or missing. Even signals that are not directly related to the activity of the heart, such as the EEG, may be contaminated with cardiac spikes that can be used for ameliorating the detection.

There already are heartbeat detectors implemented in software and publicly available for most of the individual physiological parameters that are affected by the electrical activity of the heart (e.g., GQRS for ECG [22] and WABP [23] for BP and ABP, [24] for PPG, and [25] for EEG, to name a few). However, combining the manifestations of the same beat across various signals to obtain a more robust identification requires some fusion algorithm. This is a complex task which is the subject of very active research. In ICUs and other clinical applications, robust detection from information fusion of several signals may reduce the number of false alarms, therefore increasing the confidence of health professionals in the monitoring system. Furthermore, some of the analyses carried out from the heartbeats (such as heart-rate variability analysis) are greatly affected by the loss of even a small number of beats [26]. Hence, the interest in obtaining heartbeat detections as accurate as possible grows.

The Physionet/Computing in Cardiology Challenge held in 2014 [22] and the subsequent follow-up [27] boosted the research in ECG-based fusion for heartbeat detection. Furthermore, the data provided with the challenge is a great contribution for assessing the performance of new algorithms. Since then, new techniques that aim to take advantage of fusion have been presented. In this paper, we review and compare the most important approaches for improving the reliability of single- and multi-lead ECG detectors by fusing the information extracted from this parameter with the information extracted from other simultaneously recorded physiological parameters.

The paper is organized as follows: Section 2 describes the main databases related to ECG heartbeat detection that contain multiple types of physiological signals. In Section 3, the most important heartbeat detection techniques that exploit multi-modal information fusion are described. This section has been divided into signal selection (Section 3.1), signal preprocessing (Section 3.2), feature extraction (Section 3.3), signal quality assessment (Section 3.4), detection and delay correction (Section 3.5), and fusion (Section 3.6). Section 4 describes the evaluation metrics used to measure the performance of the different techniques presented in the reviewed papers; Section 5 presents these metrics for the different techniques and discusses these results. Finally, Section 6 concludes the paper.

## 2. Databases

Although there exist many databases that contain single- and multi-lead ECG recordings along with the corresponding heartbeat labels (e.g., MIT-BIH Arrhythmia [28,29], LTST [29,30], MIT-BIH Noise Stress Test [29,31], European ST-T [29,32], and ECG-ID [29,33], among others), there is still a lack of databases that integrate ECG and other physiological signals in a single database. This section describes the main multi-modal databases found in the literature that combine ECG recordings and other physiological signals. These databases, which are available in Physionet [34], constitute an invaluable resource for research in multi-modal information fusion for heartbeat detection.

### 2.1. Physionet 2014 Challenge Database

The most relevant database related to heartbeat detection from multi-modal data up to 2014 is the one released by Physionet in the challenge held that year [22]. It includes ECG, BP, arterial line (ART), pulmonary arterial pressure (PAP), RESP, and EEG signals, among others. Data was split into training data, which is typically used for technique design, and three different sets of test data, named test phase I, test phase II, and test phase III, with which the actual technique performance can be tested. Data typically contains 10-min long recordings from human adults, though a few records contain less than 10 min; 95% of data were obtained from healthy patients, and the rest belong to human adults who suffer cardiac problems. Each record contains between four and eight signals (an ECG lead is always present). The sampling rate is not homogeneous along records: in training data, the sampling frequency of the signals is 250 Hz, and it varies between 10 Hz and 1000 Hz in test data. However, it must be noted that the sampling frequency is the same for all the signals corresponding to a given test recording. Labelling for the training data was carried out by experts, who annotated the QRS complex positions, though a few of them (four) were deemed to have been incorrectly annotated as heartbeats after further analysis. The training data can be downloaded via the web. Table 1 shows the number of records of each data set in which each of the most common signals is present, as well as their sampling frequency and duration.

### 2.2. Physionet 2014 Follow-Up Challenge Database

A few months after the challenge finished, the follow-up challenge 2014 database was released in 2015 by Physionet [27]. Nowadays, it is the most important database related to heartbeat detection from multiple physiological signals, among which an ECG lead is always present. It is divided into training data and test data. Training data consists of the challenge training data plus 100 additional records from the challenge test data (200 records with 151,032 heartbeat labels in total) and can be downloaded via the web. Test data consists of 210 records with 152,478 heartbeat labels. The records contain different types of signals, which are shown in Table 2 along with the number of records containing each type of signal.

### 2.3. MIMIC Database

This public and free-access database comprises data obtained from more than 90 hemodynamically unstable ICU patients at Boston’s Beth Israel Deaconess Medical Center from 1994 to 1996. It contains 100 records from patients who were monitored during 200 days, and their duration varies between 24 and 48 h. Each record was split in 10-min lengths for further processing. Hewlett Packard (HP) Merlin monitors were employed for data recording in different ICUs. Table 3 summarizes the information of the most relevant signals for heartbeat detection that are present in the database. All the records contain three ECG leads (sampled at 500 Hz), along with four or five additional signals (each sampled at 125 Hz) or two ECG signals and six additional signals. These additional signals include ABP, RESP, and pulse oximeter. PAP, central venous pressure (CVP), and fingertip plethysmograph (PLE) were also acquired for some records. Heart and respiration rates; oxygen saturation; blood temperature; inspired minimum and end-tidal ${\mathrm{CO}}_{2}$; fractional inspired oxygen; cardiac output; and systolic, diastolic, and mean arterial pressures were also recorded at intervals of 1024 s. Annotation files for each record are included as well. Each annotation file includes the following information: heartbeat labels; changes in the patient’s status; changes in the functioning of the monitor (though not for all the records); and ABP, PAP, CVP, and PLE signal annotations for those records in which the corresponding signals exist. The database is provided via CD-ROM. More information about this database can be found in References [29,35].

### 2.4. MIMIC II Waveform Database

This database was released in April 2011 and comprises public and free-access data obtained from adult patients in ICUs at Boston’s Beth Israel Deaconess Medical Center from 2001 to 2007. Data includes both physiological signals and minute-by-minute trends.

Physiological data was obtained with the Component Monitoring System Intellivue MP-70 Philips Health Care. It includes ECG, BP, pulse plethysmogram, and RESP signals. Signals were recorded with a fixed sampling frequency (125 Hz), and trend data was updated each minute. Additionally, labelling also contains time-stamped alert information (e.g., arrythmia) for each patient. Data was stored in three different formats: WFDB (which conveys an open data format from Physionet and also agrees with the Health Insurance Portability and Accountability Act standards), HTML, and plain text. Data can be downloaded via the web upon a free registration.

Specifically, the v2.4 version of the database comprises 25,328 ICU stays from 22,870 hospital entries, which derive 2061 records containing physiological signals and 2000 additional records that are not matched with the clinical data. More information about the database can be found in References [29,36].

An extension of this database is the MIMIC III Waveform database [29,37], which contains the same signals as the MIMIC II Waveform database but differs in the clinical data of the patients.

### 2.5. Massachusetts General Hospital/Marquette Foundation (MGH/MF) Waveform Database

The Massachusetts General Hospital/Marquette Foundation (MGH/MF) Waveform database contains hemodynamic and electrocardiographic signals of stable and unstable patients in several environments: ICUs, surgery rooms, and laboratories for heart catheterization. The database contains recordings from 250 patients, of which the length varies from 12 to 86 min, being in most of the cases one hour long. Each recording typically includes three ECG leads, arterial pressure, PAP, CVP, respiratory impedance, and airway ${\mathrm{CO}}_{2}$ signals. Some recordings also include intra-cranial, left atrial, ventricular, and/or intra-aortic-balloon pressure signals. Additional information regarding ECG calibration, zero pressure, calibration pressure, and pressure/catheter frequency response tests is also included.

The original signals were recorded with 1440 Hz as the sampling frequency with an 8-channel instrumentation tape, then digitized at twice real time, and then downsampled to 360 Hz. Each recorded signal also includes an annotation file (in .ari format) with heartbeat and event labels. The database is distributed via CD-ROM. More information about this database can be found in References [29,38].

### 2.6. IMPROVE DL Database

This database is encompassed within the Biomed-1 program of the European Union, which aims to develop biomedical signal processing and recognition methods dealing with vital-tissue oxygen delivery. Data was collected in the Department of Intensive Care, Kuopio University Hospital in Finland during 60 days continuously. Data includes 59 records, each with a typical duration of 24 h, from 50 patients that suffer hypovolaemia, cardiac problems, high flow state, and oxygen-content-related problems. Signals were acquired with a Datex AS/3, Datex-Engstrom Instrumentarium Corp., Helsinki, Finland monitor. The database includes different types of physiological signals with different sampling frequencies: 2-channel ECG (100 Hz), systemic arterial pressure (SAP) (50 Hz), PAP (50 Hz), CVP (50 Hz), ${\mathrm{CO}}_{2}$ (25 Hz), airway oxygen concentration (25 Hz), airway flow (AWF) (25 Hz), and airway pressure (AWP) (25 Hz). The data was stored using the .edf (European Data Format) format. This database also includes some signal preprocessing steps before storage: ST calculation, low-pass filters, and median filters. Annotations have a 1-min resolution.

Additionally, seven recordings from five male patients (19–78 years old) that suffer hypovolaemia, cardiac problems, sepsis (evolved into high blood-flow state), and gas exchange abnormality were also carried out. These recordings contain 2-channel EEG signals (100 Hz), ECG (100 Hz), SAP (50 Hz), CVP (50 Hz), ${\mathrm{CO}}_{2}$ (25 Hz), and AWP (25 Hz), which were also preprocessed from low-pass and median filters before storage. Data is provided via CD-ROM. Table 4 summarizes the information of the signals present in the database. More information about it can be found in Reference [39].

### 2.7. PhysioUnicaDB Database

This database consists of ECG and EEG signals obtained from 22 healthy subjects in the Neurology Department of the Hospital of Cagliari in Italy. The BrainQuick system developed by Micromed has been used for signal acquisition. It consists of 64 electrodes located mainly in the scalp of the subject but with two of them in the wrists. During signal recording, patients were told to close their eyes, to stay awake, and to reduce eye movements. They were carried out in a silent room with 1024 Hz as sampling frequency and then downsampled to 256 Hz. Signal duration varies from one recording to another, with at least two min per signal. The recordings were then segmented into 5-s intervals for data storage. A preprocessing step which consists of baseline removal was also carried. Data was stored in .edf format file. More information about this database can be found in Reference [40].

### 2.8. MIT-BIH Polysomnographic Database

The MIT-BIH Polysomnographic database comprises 18 recordings of several physiological signals carried out while the patients were sleeping to monitor chronic obstructive sleep apnea and to test whether constant positive airway pressure is beneficial in the treatment of the disease. They were carried out in Boston’s Beth Israel Hospital Sleep Laboratory. Recordings amount to more than 80 h, each of them with a length between 2 and 7 h. Annotated ECG, invasive BP, EEG, and respiration (in the majority of the cases, from a nasal thermistor) signals are always present for each recording. Some recordings also include another respiratory signal (derived from inductance plethysmography) and some of these two signal groups: EOG/EMG signals (measured at the chin) and cardiac SV signal/earlobe oximeter signal. The sampling frequency in all the cases is 250 Hz. The database is distributed via CD-ROM. Table 5 summarizes the information of the signals present in the database. More information about it can be found in References [29,41].

## 3. Heartbeat Detection from Multiple Physiological Signals

**Figure 1:**
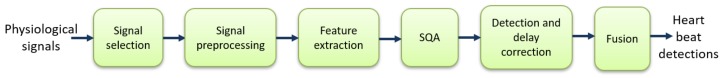
Typical steps of heartbeat detection: “SQA” stands for signal-quality assessment.

This section presents the main approaches of the literature that employ fusion of information extracted from the ECG and from other physiological signals for heartbeat detection. The standard architecture of a multi-modal heartbeat detector can be seen in Figure 1. The subsections of this section are organized according to the steps shown in this figure.

### 3.1. Signal Selection

Signal selection is a necessary stage when different types of physiological signals can be used for heartbeat detection. On the one hand, the complementary information that some of them convey could improve performance through the fusion of the information each provides. On the other hand, selecting multiple signals that convey the same or similar information may not outperform the heartbeat detection performance and even could reduce it due to the limited amount of data (theoretically, with infinite amount of *training* data, an algorithm should reject the *bad* data, but in a real scenario, there is only a finite amount of data).

**Figure 2:**
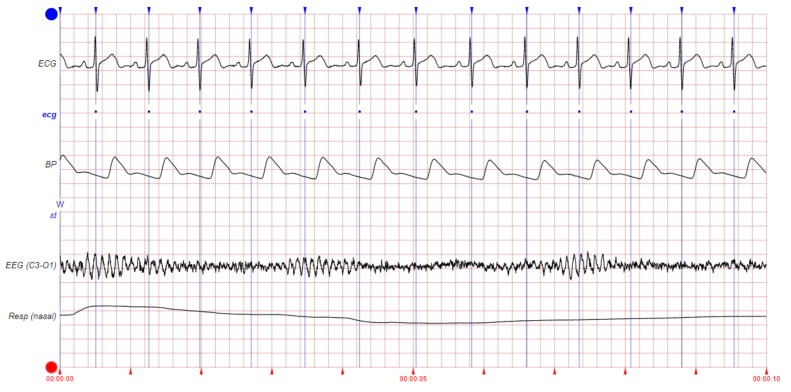
Examples of signals of interest for heartbeat detection: Blue vertical lines show the heartbeat annotations. This figure corresponds to slp04 record from MIT-BIH Polysomnographic Database.

We shall classify large amounts of physiological signals that can be recorded regarding heartbeat detection into the direct signal group and the indirect signal group. The direct signal group contains the signals that are directly related to cardiac activity (e.g., ECG, ABP, BP, PPG, SV, PAP, SAP, CVP, and ballistocardiogram (BCG), among others). The indirect signal group is composed of the signals that are not related to cardiac activity but that are influenced by such activity, and therefore, they could provide useful information for heartbeat detection (e.g., EEG, EMG, EOG, and general pressure (PRESS), among others). Figure 2 shows some examples of these signals of interest. It can be appreciated that some physiological signals (e.g., BP) have their peaks delayed with respect to the ECG peaks. This should be taken into account during heartbeat detection to correct the position of the beats (see Section 3.5.3).

Among the direct group, the ECG records the electrical activity of the heart. Therefore, it is the most suitable signal for heartbeat detection, since the signal is directly generated by the phenomenon of interest (the heartbeat). The PPG measures the volumetric change of the heart by measuring light transmission or reflection. When the heart contracts, a pulse of blood is sent into the arteries of the body. By detecting peaks in the amplitude of this signal, those pulses and, hence, the heartbeats can be detected. The SV is the volume of blood pumped from the left ventricle per beat. It is computed by subtracting the volume of the blood in the ventricle at the end of a beat from the volume of blood just prior to the beat. The BCG signal, which emerges from a minuscule motion of the human body in response to the recoil forces of the cardiac ejection into the vascular system, also comprises heartbeat detection information.

The SAP is the primary determinant of cerebral blood flow. It is computed from the cardiac output and, hence, is related to heart function. The BP signal relates to the pressure of the blood within the circulatory system. When the heart beats, it pumps blood around the body to give it the energy and oxygen needed. As the blood moves, it pushes against the sides of the blood vessels. The BP is the strength of this *pushing*. After the pumping of blood produced by a beat, there is an increase in BP, followed by a decrease until the next blood pulse (beat) arrives. The specific moment at which the maximum pressure is reached after a beat depends on the distance between the heart and the point of the body in which the pressure is being measured. The more distance, the longer the delay in the arrival of the peak of the pressure wave. The different pressure-related signals (i.e., ABP, BP, CVP, PAP, and PRESS) measure pressure in different parts of the body. Through the count of the number of local maximums (or minimums) that occur in the BP, it is possible to establish a patient’s heart rate (see Figure 2).

Among the indirect signal group, the EEG records the cortical electrical activity of the brain from the scalp. The relatively high electrical energy of the cardiac activity causes EEG artifacts, contaminating the EEG signal with QRS complexes (see Figure 2). The EOG signal measures the electrical activity of the eyes from the corneo-retinal standing potential that exists between the front and the back of the human eye. The EMG records the electrical activity of the skeletal muscles by measuring the electrical potentials generated in them. The EOG/EMG signals are also contaminated with the electrical potentials generated by the QRS complexes (see Figure 2). Therefore, by looking for those artifacts, the QRS complexes can be identified over the EEG/EOG/EMG signals, making them potential sources of information about the heartbeat.

Table 6 presents the signals that have been employed in the reviewed papers. Most of the proposals for heartbeat detection employ the ECG and ABP signals, since these are directly related with cardiac activity [42,43,44,45,46,47,48,49,50,51,52]. Other authors have added some other signals to that group: PPG signal [53,54]; SV and PPG signals [55]; and EEG, EOG, and EMG signals [56,57].

The use of ECG and BP signals has also been extensively studied due to their direct relationship with cardiac activity [58,59,60,61,62,63,64,65], from which other approaches that integrate up to 3 additional signals have also been presented: SV [66], EEG [67,68], and EOG [69,70] signals; EOG and EMG signals [71]; SV and EOG signals [72]; EEG, EOG, and EMG signals [73]; and SV, EEG, and PPG signals [74].

The combination of ECG and SAP signals has also been studied [75,76], and the combination of the most commonly used signals (ECG+BP and ECG+ABP) with some other signal/s as well [77,78,79] has been studied. In particular, the pulmonary arterial pressure (PAP) signal is added in References [77,78], and SV and central venous pressure (CVP) signals are added in Reference [79] to those presented in References [77,78]. The most extensive signal set composed of ECG, BP, ABP, SV, PAP, CVP, PRESS, PPG, EOG, EEG, and EMG signals is employed in Reference [82], and ECG, BP, ABP, PRESS, PPG, and SV are used in Reference [81]. BCG along with the ECG has been employed in Reference [80].

### 3.2. Signal Preprocessing

Signal preprocessing is often needed to improve the quality of a signal for the subsequent stages. A summary of the signal preprocessing techniques is presented in Table 7. Low-pass filters [44,45,57,71,73,80] and band-pass filters [44,55,56,59,65,79,81] are widely used for ECG preprocessing. Different cutoff frequencies have been proposed for low-pass filtering, including 40 Hz for the ECG and BP signals [57,73]; 35 Hz for the ECG, BP, EOG, and EMG signals [71]; 20 Hz for the BCG signal [80]; and 16 Hz for the ECG and ABP signals [44,45]. Regarding the band-pass filters, cutoff frequencies spread in the range 0.5–10 Hz for the ABP signal [44], and 0.5–80 Hz for the ECG signal [65]. In Reference [56], different cutoff frequencies are employed depending on the signal (5–40 Hz for ECG, 5–55 Hz for EEG, 10–25 Hz for EOG, and 5–15 for EMG). In some works, the cutoff frequencies of the band-pass filtering are computed from the percentiles of the RR intervals obtained from the QRS detection in the BP signals [59] and from the d2, d3, and d4 coefficients of the wavelet transform in the ECG signals [79]. High-pass filters have also been used in Reference [80], with cutoff frequencies of 1 Hz for the ECG signal and 0.5 Hz for the BCG signal.

Baseline wander is a key point when processing ECG signals. This is typically caused by electrode-related issues, movement, and respiration of the person [83] and affects the low-frequency components (below 0.5–0.6 Hz) of the ECG signal [84] and, in general, of any signal that contains heartbeat detection information. From the signal recording perspective, baseline wander causes the signal to shift from its normal base. To address this, baseline wander suppression has been widely used: in Reference [65], a two-order smooth filter was applied to the ECG and BP signals. In Reference [44], 0.5–10 Hz band-pass filtering has been used for the ABP signals. Approximation coefficients of the wavelet transform are used in References [67,85] for the ECG signals. From all the filters that are applied in cascade in Reference [63], the last high-pass filter used in the quadratic spline approach in that work is used for ECG and BP signals. Band-pass filtering with cutoff frequencies of 5–40 Hz has been employed for ECG signals in Reference [56]. In Reference [57], a moving median filter is used. In Reference [78], the convolution-based filtering serves as baseline wander suppression for ECG, BP, ABP, and PAP signals. Cascade median filters are used in Reference [52] for the ECG signal. In Reference [79], the d1 coefficients of the wavelet transform are removed for ECG, BP, ABP, PAP, SV, and CVP signals.

Power line interference can also introduce noise in the signal recordings, which causes variations in signal parameters (e.g., amplitude and duration, among others) and may lead to diagnostic errors. This noise occurs at 50/60 Hz frequencies. Notch filtering, aiming to remove this power line interference, has been employed in References [65,80].

Other types of filters such as moving average [61,66], median [73], mean [57], anti-aliasing [70], quadratic spline (from low and high-pass filters) [63], and convolution-based filtering (OWN) [78] have also been used. Wavelet transform has also been employed in Reference [47].

To unify the sampling frequency of the input signals, which enables a meaningful frequency-based signal analysis, downsampling [61,71,80,81] and resampling techniques [43,52,63,70,72] are also common preprocessing steps. Signal normalization has also been considered in References [52,61,63,72,78,79].

### 3.3. Feature Extraction

Feature extraction aims to produce the most discriminative set of features from the incoming (or preprocessed) signals so that heartbeat detection is enhanced. Although not all the proposals integrate a feature-extraction process (where not, the detection algorithm is largely based on the raw data of the incoming signal or the data after signal preprocessing), some feature extractors have been proposed. These can be classified in different categories depending on the type of features obtained: time-based approaches, frequency-based approaches, and time–frequency-based approaches. Table 8 presents a summary of the feature-extraction methods employed in the reviewed papers. It must be noted that any feature of Table 8 can also be used for heart-rate estimation due to the tight relationship between heart-rate estimation and heartbeat detection. Heartbeat detection enables heart-rate estimation, since once all the heartbeats from an incoming signal have been detected, the heart rate can be obtained by, for example, averaging the number of heartbeats detected within a time window. However, heart-rate estimation cannot be used for precise heartbeat detection, since precise timing information related to the occurrence of each beat is missed. Therefore, whereas heartbeat detection can be used for systems that need the precise temporal location of the beat (e.g., arrhythmia classification and heart rate variability analysis, among others), heart-rate estimation cannot.

#### 3.3.1. Time-Based Feature Extractors

U3 transform [86,87] has been employed in Reference [70] for the ECG signal and the first derivative has been employed for the rest of the signals as the feature extractor. The U3 transform is defined in a window-basis as follows:

$$U3_{i}=\sum_{k=2}^{L}{\left(x_{i+k}-x_{i+k-2}\right)}^{2},$$

where *x* is the input signal, *i* is the current window, and *L* is the window size in samples.

Lag-adaptive short-time autocorrelation function [88], the average magnitude difference function [89,90], and the maximum amplitude pair function, which considers the amplitude of the signal, have been used for signal representation in Reference [81].

A symbolic discretization method [91] from the original continuous time series that represent the ECG and BP signals has been presented in References [58,72]. It produces a set of subsequences as features. In Reference [72], dimensionality reduction from piecewise aggregate approximation and signal discretization from a Gaussian distribution for the specified alphabet size are added.

Slope-sum functions from the first derivative of the input signals to produce the feature vectors, which are then normalized and downsampled, were used in References [44,45] as signal representation. A derivative-based approach as feature extraction in the SAP signal is also employed in Reference [75]. Derivative methods will be explained in more detail in Section 3.5.

#### 3.3.2. Frequency-Based Feature Extractors

Power spectral density (PSD) was used in References [74,82], and Fast Fourier Transform (FFT)-based frequency features were employed in Reference [71] as signal representation.

On the other hand, integer-multiplier digital filters [92,93] based on slope-sensitive and peak-sensitive sampling-frequency adjustable band-pass filters were employed in References [62,77], to which Gaussian low-pass filters with first- and second-order derivatives followed by a moving average low-pass filter were added in Reference [77] for feature extraction.

#### 3.3.3. Time-Frequency-Based Feature Extractors

The wavelet transform was used in References [47,49,63,65], to which pulse score for scale determination [94] was added in Reference [67] to compute the features for the ECG signals.

Feature extraction in Reference [68] relies on the power of the QRS complex computed in a predefined frequency range and kurtosis for the ECG signal and on the pressure ranges and average derivative for a cycle for the BP signal [95] to compute the signal quality index and the maximum and minimum of the BP signals for BP signal representation.

### 3.4. Signal-Quality Assessment (SQA)

It is well known that signal combination can lead to better performance if the signals are properly recorded (i.e., in absence of noise effects and enough sampling frequency, among others) and if they provide complementary information. On the other hand, noisy signals can dramatically reduce beat detection performance due to its inherent bad quality. Therefore, signal-quality assessment plays an important role when multiple physiological signals are used for heartbeat detection, so that those lower quality ones can be automatically rejected, either before or after heartbeat detection. Table 9 presents a summary of the SQA approaches employed in the reviewed papers, which are described in detail in the following subsections.

#### 3.4.1. Statistical-Based Signal-Quality Assessment

Fisher’s g-statistic [96,97,98] is employed in Reference [59] to determine the BP signal quality based on the periodicity of the given window signal. That statistic is defined as the ratio of the largest periodogram value to the sum of all the periodogram values over 1/2 of the frequency interval (0, Fs/2, with Fs as the sampling frequency). Larger g-values mean better BP signal quality.

The SQA approach presented in Reference [65] is based on a correlation method in which the cross-correlation between the signal segments with the signal templates is computed. The quality index assigned to each signal is the median of all the segment correlations.

#### 3.4.2. RR Interval-Based Signal-Quality Assessment

The histogram distribution of the intervals of the R-peaks that comprise the output of the detectors for the different signals has been used in Reference [57] to assess signal quality.

In Reference [66], the number of annotations in a signal segment, the variance of the peak distance (i.e., of the RR interval), the signal variance, and the peak distance (i.e., the RR interval length) have been used as features to compute the signal quality.

An approach based on the deviation of the heartbeat from the rhythm of heartbeats and on the probability of the heartbeat being matched with the given deviation has been proposed in Reference [56].

The SQA approach presented in Reference [79] is based on three different stages: in the first stage, the quality of ECG and BP (or ART) signals is measured by checking compatibility of all the detected heartbeats on these two signals. In the second stage, a quality level is assigned by heartbeat comparison in every possible window. In the last stage, which only runs when incompatibilities occur in the second stage, quality is assigned to the ECG signal by a sample dispersion-based approach.

Heart rate variability [26] as a measure of the signal quality has been used in Reference [54]. This is computed from the standard deviation of the eight most recent heartbeat intervals normalized by the mean of them.

In Reference [55], a signal-quality measurement called fusion regularity (FREG) considers the most regular of a set of RR interval time series for consecutive windows so that if a time series contains less than three detections within a given window (15 s), this is considered bad quality.

#### 3.4.3. Signal-Abnormality Index (SAI)-Based Signal-Quality Assessment

SAI [95] considers BP/ABP signals as bad quality in cases where the signal values are not within reasonable physiological ranges. They include pressure ranges, systolic blood pressure (SBP), diastolic blood pressure (DBP), mean arterial pressure, duration of each heartbeat, heart rate, and the mean of the negative slopes.

ABP signal quality in References [47,50,55] and BP signal quality in Reference [68] are computed from the SAI, which integrates the pressure ranges and the average derivative for a cycle for the respective signals. The SAI is also employed to assess the ABP signal quality in References [42,46].

#### 3.4.4. Detector Level Agreement-Based Signal-Quality Assessment

A number of algorithms that detect each potential peak over multiple signals has been employed in Reference [43] as signal-quality index. Similarly, in Reference [55] (fusion signal quality indices (FSQI)) and in Reference [47,50], an ECG signal-quality approach based on the level agreement of two R-peak detectors has been proposed.

In Reference [44], the quality of ECG signals is assessed based on two ECG peak detections algorithms [100,101]. Then, it is checked whether their detections are matched or not. Signal segments for which the F1 score is 1 are considered as good quality. The same approach of has been employed in Reference [45], but it has also been extended to ABP quality assessment. For ABP signal quality assessment in Reference [45], it is based on two independent ABP peak detectors following the same F1 score-based approach of Reference [44].

#### 3.4.5. Discrete Wavelet Transform (DWT)-Based Signal-Quality Assessment

Two quality indexes for the ECG and BP signals based on the noise estimation from the coefficients of the second level of detail output by the DWT have been proposed in Reference [64]. To do so, each segment in which the distribution of noise is lower will be assigned a higher quality index. A second index is derived from the periodicity of the detected heartbeats in each segment. Both indexes are summed to create the final signal-quality index for each segment.

The energy of the detailed coefficients of the DWT has been used in Reference [49] as a signal-quality metric (the lower this energy is, the higher the quality is).

#### 3.4.6. Other Signal-Quality Assessment Approaches

In Reference [44], the ABP signal quality is obtained by integrating a band-pass filter along with two different signal-quality indexes. One of these relies on the periods of saturation of the signal to a maximum or a minimum value so that, if these periods are above a predefined threshold, the quality is set to be bad. The other index considers the kurtosis of the distribution of each segment. The multiplication of these indexes provides the ABP signal quality.

The upper and lower boundaries and the RS interval length from RS slope detection have been used in Reference [73] for SQA.

Signals with a variance in the delay values after heartbeat synchronization above a predefined threshold are considered as bad quality in Reference [74].

The relative power of the QRS complex and the kurtosis distribution have been proposed in Reference [68] for SQA.

In Reference [82], the Hjorth’s mobility is used for SQA so that the signal with the lowest values is assigned the best quality.

The sample entropy [99] has been used in Reference [48] to compute the quality of each signal segment.

In Reference [55], the U3 transform [86,87] is applied to the EEG/EOG signals. The U3 amplitude of those spikes that occur at the same time over different signals is compared with the rest of the U3 spikes. If the amplitude of the former set is larger than the latter, the EEG/EOG signals are used for heartbeat detection.

### 3.5. Detection and Delay Correction

This stage is the *core* of the heartbeat detection, since its goal is to detect heartbeats over the available signals and to compensate for any delay that could exist between the heartbeat instant of detection over a given signal and the actual instance of occurrence (such as it happens with the BP signal).

Most of the reviewed techniques first try to detect heartbeats separately in each of the signals of interest. The main advantage of this approach is that well-tested detection algorithms may be used. Table 10 summarizes the detection algorithms that are employed as part of different fusion proposals. This table only includes well-known algorithms (or specific implementations, if possible) and not novel proposals presented as part of the fusion methods. From this table, it follows that GQRS and WABP are the most commonly employed algorithms for heartbeat detection in ECG and BP/ABP signals, respectively. Although some papers do not directly use the methods from Table 10, most of them do reuse some of their key ideas, usually borrowed from the signal processing literature, and even apply those methods to EEG, EOG, and EMG signals (e.g., Reference [57]).

#### 3.5.1. Peak Enhancing

In general, most algorithms try to transform the raw signals into other signals where the presence of the waveforms is signalled with a strong peak. The resulting signals are suited for applying a peak detection algorithm, along with more modern approaches based on Bayesian methods, machine learning, and data mining. We may distinguish three main strategies among these algorithms: *derivative-based detectors*, *template matching*, and *others* (see Table 11).

##### Derivative-Based Detectors

Derivative-based detection (sometimes also referred to as slope-sum methods, although some authors may be referring to the specific proposal of Reference [23]) is the most broadly used method for enhancing the peaks of the signals. Derivative-based detectors have been used in References [66] (for SV signals), [69] (BP, SV, and PPG), [44] (ECG and ABP), [70] (BP, SV, and PPG), [78] (ECG, BP, ART, and PAP), and [75] (SAP), among others. Most of these derivative-based detectors are defined as follows:

(1) $$y_{i}=\sum_{k=0}^{L}h_{k}\Delta^{\left(j\right)}x_{i-k},$$

where *L* is the length of the filter $h_{n}$ and $\Delta^{\left(j\right)}x_{n}$ is an approximation to the *j*th derivative of the input signal $x_{n}$. Typical values for *j*th are 1 (first derivative) or 2 (second derivative). Examples of $\Delta^{\left(1\right)}x_{n}$ and $\Delta^{\left(2\right)}x_{n}$ may be as follows:

$$\Delta^{\left(1\right)}x_{n}=x_{n}-x_{n-1}\;\Delta^{\left(2\right)}x_{n}=x_{n}-2x_{n-1}+x_{n-2},$$

although other definitions are possible. The filter $h_{n}$ is usually a moving average filter, and it is usually employed to compute a smooth version of the derivative’s energy (acting like a low-pass filter). Note that $h_{n}$ is defined as a causal filter in Equation (1), although this is not necessary.

The U3 transform introduced for QRS detection in References [86,87] can be considered a derivative-based approach. The U3 transform is applied to other signals besides the ECG in References [69] (EEG/EOG) and [70] (EEG/EOG).

##### Template Matching

Template matching has also been used as a method for detecting the presence of a reference pattern in the signal. This is usually achieved by cross-correlating the reference pattern with the signal of interest. Then, annotations are usually found by examining the cross-correlation signal. Template matching is employed in References [53] (ECG, ABP and PPG), [65] (ECG and BP), [71] (ECG, EOG, EMG, and BP), and [72] (ECG and BP), although there are significant differences between them, especially referring to the generation of the template pattern or its intended use.

The proposal from Reference [53] differs from the others since it uses a variant of Dynamic Time Warping (DTW) for detecting the heartbeats. Since this algorithm performs detection and fusion at the same time, it will be described in Section 3.6.4.

The proposal from Reference [65] uses template matching not only for removing pacemaker pulses but also for removing incorrect R-peaks and for assessing the quality of both ECG and BP signals. The template of pacemaker pulses is computed by manually annotating pacemaker records. The template for the R-wave is built from a set of preliminary annotations (obtained through a Continuous Wavelet Transform (CWT), the peak detector from Reference [117], and Linear Discriminant Analysis (LDA)), and the patterns for quality assessment are made from short segments (24 s) of the record being analysed.

In Reference [71], preliminary annotations are generated using the most salient peaks in each signal from the FFT-based features. Then, averaged shapes (the templates) are computed from the area that surrounds each preliminary annotation.

In Reference [72], several algorithms based on template matching are compared with a baseline detector (based on GQRS and WABP). We may distinguish three main approaches. In the *full beat template* approach, a different template is created for each of the heartbeats of the Physionet 2014 challenge test set. In the *clustered beat templates*, K-means is applied to all the test set heartbeats to reduce the number of beat templates. Finally, *statistical templates* are also considered. In this approach, the templates consist of summary statistics instead of the samples of the waveform.

##### Other Peak-Enhancing Techniques

Among the *other* methods that try to facilitate the use of a peak detector, it is worth highlighting envelope functions (a smooth curve outlining the extremes of an oscillatory function), used in References [61,71,74,77,82]; enhancing peaks through the wavelet transform [63,65,79]; morphological filters [62,77]; applying range filters (they compute the difference between local maxima and local minima) [61]; directly thresholding the signal (specially, the ECG, which is “peaky” in nature) [79]; T-wave suppression filters [80]; and using adaptive filters such as in Reference [67] (in which the ECG is filtered to try to remove EOG peaks; the output of the filter is used for QRS detection).

#### 3.5.2. Peak Detection

Peak detection involves searching for local maxima, usually by direct comparison with neighbouring values. For a robust detection, the local maxima are required to meet some conditions. Common requirements include a minimum height (this removes spurious peaks), a minimum prominence (a measure of how much a peak stands out from the neighbouring), and a minimum distance between two consecutive peaks (usually between 200–300 ms for heartbeat detection, see References [57,78,82]). In Reference [54], a simple peak detector implemented as a moving window with 0.3 s finds a peak if the middle value of this window is the maximum value of the overall window. Although some proposals [78,82] implement their own peak detectors from convolution-based filtering [78] and envelope functions [82], most researchers prefer to use the tools included in the programming language of their choice (such as MATLAB’s *peakdetect*) or specialized software [117]. It must be noted that some proposals directly use the peak detection functions without any preprocessing [66].

Detection algorithms are not restricted to classical signal processing algorithms, but it is increasingly common to use other techniques, specially from the field of machine learning or Bayesian statistics. The different approaches for peak detection are summarized in Table 12.

In Reference [81], a Bayesian approach able to fuse ECG with some other pulsatile functions is proposed (it selects, in this order, one of the following signals: BP, ABP, ART, PRESS, PLETH (PLETH refers to the same PPG signal but with another tag name),PPG, or SV). Three different estimates of the interbeat intervals are obtained using the set of features presented in Section 3.3. These estimation algorithms are run on moving windows to find the interval estimations in every signal. The final interval estimation is obtained by combining the three estimates through a Bayesian approach. GQRS and WABP tools from the WFDB toolbox are employed to obtain another estimate of the correct annotation locations. These locations are compared with the mean estimated interval from the Bayesian approach so that annotations that appear in near positions comprise the final annotation list.

Machine-learning approaches have also been proposed for multichannel heartbeat detection [52,63]. In Reference [63], two different classifiers are tested: Neural Networks (NN) and the XGBoost gradient boosted tree (BT) algorithm. The classifiers are trained with small snippets of data, which are labelled as 1 if there is a heartbeat in the middle of the snippet (and otherwise as 0). Therefore, the output of the classifiers may be interpreted as the probability of detecting a heartbeat in the middle of the snippet.

Finally, it is worth mentioning approaches based on data mining algorithms: in Reference [58], heartbeat detection methods based on a sequential pattern framework are proposed. To do so, the ConSGapMiner algorithm [119] extracts subsequences that may correspond to a heartbeat from the signal feature representation (see Section 3.3). Finally, all the generated subsequences are ranked and subsequently used to hypothesize the annotations.

The Bayesian approaches from References [44,45,50,51] and the machine-learning method from Reference [52] merge the detection and fusion steps into a single one, and therefore, they will described in Section 3.6.4.

#### 3.5.3. Delay Correction

There could exist a delay between the R-peak of the ECG and the associated peaks in other signals. In the case of BP, this delay is known as Pulse Transit Time (PTT). These delays should be taken into account to fuse the annotations from several signals. Most algorithms try to tackle the issue by estimating the delay and by correcting it before the fusion algorithm. The main differences between the different proposals arise at the delay estimation step. A summary of these proposals is shown in Table 13.

The simplest approach is to use a fixed delay value for each signal taken from the literature (for example, 200 ms for the BP signal) [52,64,78,79]. Some authors also use constant delay values for all the records (and each signal), although they estimate the values from the data [45,75,81].

The delay is most usually corrected in a patient-specific basis [47,48,49,55,56,57,66,69,70,73,74,82]. The estimation is usually based on the mean, median, or mode of the delays between the annotations, although some authors use the cross-correlation between the annotations and the signal shifted [69,70]. Some of these algorithms try to focus on clean segments to achieve an accurate estimate [47,55,66], and most of them use constant default values taken from the literature for those cases where the estimation cannot be done [47,55,66,69,73] or where it yields unreasonable physiological values [69,70]. In Reference [74,82], an even more drastic approach is taken: if the standard deviation of the delay values is too high, the signal is discarded.

Although the delays are known to vary between consecutive contractions, there is a good reason for using a constant value for the delays. Besides the simplicity of this approach, the variance of the delay times is usually negligible compared to the margin that is usually permitted for an annotation to be considered correct (150 ms in the Physionet Challenge 2014) [79].

Even so, some algorithms estimate a dynamic delay time for the signals, accounting for the variation between successive contractions. The most broadly used method consists of smoothing the delays between QRS peaks and the peaks from the signal of interest, either by using a moving average filter [60,61,67] or a Hampel filter [120,121], which has been used in Reference [42]. These filters help in eliminating the effect of wrong delays due to missing or additional annotations. The delay may also be estimated by using the correlation method in small windows [62,65,77] or by using the variance of the physiological delay between electrical and mechanical myocardial activation [76]. In addition to the correlation method, Reference [62] also proposes estimating the proper shift of the signal of interest by using a linear regression. In this case, the model predicts the proper shift using as the predictor the interpolated pulse rate of the signal of interest. Again, a default value can be used if the method predicts unreasonable values [42].

### 3.6. Fusion

The fusion stage is necessary to produce the final annotation list that contains all the heartbeats detected over the multiple physiological signals. A good fusion strategy can improve heartbeat detection by exploiting complementary information present in the different physiological signals while, at the same time, avoiding those signal intervals that have poor quality or high levels of noise.

There exist multiple strategies used to combine the annotations from the different signals. We shall distinguish four main approaches, although other classifications are possible, in particular when considering the diffuse edges between them and that some overlap exists. These approaches are RR-based methods (Section 3.6.1), signal switching (Section 3.6.2), voting (Section 3.6.3), and approaches that merge heartbeat detection and fusion into a single step (Section 3.6.4). RR-based methods rely on the beat-time information previously obtained, since the fusion is based on the time between two beats (often, between the R-peaks of two QRS complexes). This makes precise time location of the beat crucial for fusion and may present a bottleneck for improving the performance. Signal switching depends on the SQA algorithm, since the annotations that are kept in the final list are obtained by using the signal which yields the best SQA measurements. In this case, employing a robust SQA algorithm is a must. In voting approaches, each potential beat detection over a signal constitutes a vote for the final beat presence, a vote that is often weighted by some metric of the signal’s reliability and/or quality. This may be similar to the signal-switching approaches, since these also employ some kind of voting (i.e., they rely on the detection provided by the *best* signal). Techniques that combine detection and fusion in a single step borrow the ideas either from statistical methods (often Bayesian approaches or techniques based on Hidden Markov Models) or from machine learning for merging detection and fusion into a single step.

Some authors [80] have also proposed a manual fusion from the morphology and timing of the IJK complex in the BCG signal and of the R and T peaks in the ECG signal. Table 14 presents a summary of the fusion approaches used in the reviewed papers.

#### 3.6.1. RR-Based Methods

A common approach for combining the annotations obtained over different physiological signals is joining all the annotations into a single annotation list, sorting them, and combining very close annotations (usually employing a window of 150 ms approximately). Then, the RR intervals are used for detecting missing annotations (which would yield a very large RR interval) or spurious annotations (which would yield a very short one). Usually, spurious annotations are directly removed whereas missing annotations are predicted using interpolation or the mean RR interval. This approach or similar ones are used in References [42,46,57,58,59,61,64,72,73,78,79]. The process may be repeated several times until convergence [81]. It is worth discussing some of the variations of this idea.

Some proposals use advanced filtering techniques for improving the detection of outliers within the RR series. For example, in Reference [42], a Hampel filter [120,121] is used for detecting outliers, which are then interpolated using a nearest neighbours approach. On the other hand, the Hjorth’s mobility is employed in Reference [74] for estimating the number of missing annotations.

In Reference [43], a nearest-neighbour selection scheme is employed. In this way, in case the annotation is output by two or more heartbeat detection algorithms, the end time and peak value corresponding to the mode RR interval time are assigned to the given annotation, and in case the annotation is output by a single algorithm, the end time and peak value yielding an RR interval closest to the previous averaged 12 RR intervals are assigned to the corresponding annotation.

The “sandwich rule” proposed in Reference [60] states that an R-wave is valid if two conditions are met: It is the only R-wave between two consecutive ABP onsets, and each of these ABP onsets is the only onset between two consecutive R-waves. In the simplest cases, an invalid annotation (either from ECG or ABP) is corrected by using a mean QRS-BP delay. For example, if two consecutive BP onsets “sandwich” more than one QRS, the mean QRS-BP delay is used to predict proper positions of the missing QRS peaks. Since in pathological cases (such as premature ventricular heartbeats or a very noisy ECG segment) the “sandwich rule” may fail, the authors perform a sanity check to ensure that the ECG peaks are indeed QRSs. The idea is that a QRS complex will intersect a set of regularly spaced horizontal lines placed over the ECG at most six times, whereas this number will be probably larger in noisy segments. The “sandwich rule” is also applied in Reference [42].

It is worth noting that RR intervals are also used to refine the final annotation list that results after the fusion algorithm [47,59,61,63,74,78,82]. The techniques employed to that end are very similar to those introduced at the beginning of this subsection.

#### 3.6.2. Signal Switching

A recurrent idea which usually yields good performance is generating the annotations by switching from one signal to another depending on the quality index of the current segment. Usually, a hierarchy of signals is built. For example, if the ECG has enough quality, the annotations from the ECG are used, and if not, the quality of the BP is assessed; if it is good enough, its annotations are used, and if not, the signal with the highest quality among the remaining ones is used. Examples of these approaches can be found in References [47,48,49,55,57,62,65,66,68,77]. The method presented in Reference [79] simply rejects an annotation in case this belongs to a signal segment in which the physiological range values are out of a predefined interval.

#### 3.6.3. Voting

Among the voting approaches, we may distinguish between majority voting and weighted voting. In majority voting, each of the signals votes for the presence of a heartbeat in a small window. The final heartbeat location is then found by searching for a local maximum or by requiring the agreement of a minimum number of signals (usually, half plus one). Majority voting is used in References [67,74,79,82].

Majority voting can be improved by assigning different weights to the signals according to diverse criteria. We shall summarize some of the algorithms using weighted voting to illustrate possible weighting schemes.

In Reference [69,70], a majority voting technique using a Tukey window is proposed. However, different weights are assigned to the signals (according to its type), so that ECG, BP, or PPG can trigger a detection on their own. However, two simultaneous detections over SV, EEG, and EOG or a single detection over any of these signals overlapping with the location of a predicted heartbeat (using linear interpolation) are required to trigger a final detection.

In Reference [56], a majority voting fusion method integrates the information from the multiple physiological signals as follows. First, the signals are segmented into small windows and the average heartbeat signal quality index (SQI) is used to choose the best signal as reference signal. In this reference signal, each RR interval is selected and considered as a potential annotation in case it fits within some tolerance limits. All the annotations in the other signals within a window of 150 ms are considered for fusion. To that end, weights for each signal are assigned for majority voting, so that signals below a predefined threshold are considered noise and rejected in the voting. Finally, the mean temporal location computed from the annotations in the voting produces the final annotations.

In Reference [71], the annotations are created by combining all the channels (which were preprocessed using template matching; see Section 3.5) into two different new signals: the Total Correlation Response (TCR) signal, which weights all the channels according to the mean correlation between the channels and the template pattern, and the Best Correlation Response (BCR) signal, which just picks the temporal maximum across the channels. The annotations are created by ensuring that each new detection is far enough from the previous one and by switching from TCR to BCR when the quality of TCR is too low. In Reference [54], Bayesian inference that permits weighting the different signals is employed for fusion.

The work presented in Reference [76] uses the optimal fusion method proposed in References [122,123]. This decision rule combines the individual decisions of each detector, weighted according to its performance. On the other hand, *and* and *or* rules are applied in the annotations in Reference [75] to confirm or reject these. In this work, a heartbeat detection is copied to the final annotation list if it is output by the two signals of interest (*and* rule) or only by a single signal (*or* rule).

#### 3.6.4. Simultaneous Detection and Fusion

In Reference [53], all the signals are employed to build a matrix that represents a multiparameter signal in which rows represent time and columns represent the type of signal to fuse. Then, the Euclidean distance is used to compute the similarity between each new signal and predefined templates for all the signals of interest. This Euclidean distance matrix is given to a variant of DTW, named Weighted Time Warping, which looks for the annotation boundaries. This algorithm yields as the result the final annotations, and therefore, it performs detection and fusion at the same time.

In References [44,45], hidden semi-Markov models (HSMM) are employed for robust heartbeat detection. The HSMM uses two hidden states (QRS complex and non-QRS complex) and a Gaussian emission function. To estimate the state of the ECG and BP signals, features obtained from derivative-based filters are fed to the HSMM. The most likely sequence of states for the observable signals are obtained using the Viterbi algorithm. As the Viterbi algorithm provides output probabilities for each feature vector, these are then merged with the signal-quality index computed previously to output the final annotations.

The algorithm from Reference [51] proposes fusing ECG and ABP signals using a more complex Bayesian approach, which is based on two layers. In the first layer, signals are decoded in terms of states related to well-known waveform segments: *ISO* (isoelectric), *P*, *PQ*, *QRS*, *ST*, and *T* for the ECG and *SBP*, *DBP*, *Diastolic cusp*, and *Offset* for the ABP. This is achieved by modelling the waveforms as a Hidden Markov Model (HMM). The second level uses the HMM decoded states of the ECG and ABP to detect the presence or absence of a QRS segment. The authors propose two different models to make this decision. The simplest model uses a Bayesian Network (BN) to model the relationships between the three relevant random variables of the problem: the state of the ECG *E*, the state of the ABP *B*, and the classification output *C* (which is a binary random variable). Within this model, the authors test different BNs which may be broadly categorized into two categories: BNs assuming that *E* and *B* participate independently in the decision about *C* and BNs assuming that *E* and *B* are dependent on each other for deciding *C*. The second model the authors propose is justified by the observation that consecutive states of the signals are correlated in time. This information can be incorporated in a model using state transitions, which results in Dynamic Bayesian Networks (DBNs). Just like with BNs, the authors test different transition models that show correlations between *E* and *B* or not.

Yet another example of a Bayesian approach is Reference [50], which fuses ECG and BP using a generative model that captures a simplified understanding of the heart rhythm. The graphical model is a dynamic Bayesian network that relates hidden state variables (such as heart rate or a binary variable indicating if a BP peak is present) with both observations obtained from the GQRS and WABP algorithms and signal quality indices. The hidden states (including *ECGPeak* and *ABPPeak*) are learned by applying a particle filtering to the signals, which are first split into 25-ms windows. The hidden states are then used to annotate the signal. Specifically, the annotations correspond to timestamps where enough particles are in a state of *ABPPeak*. The position of the annotation is corrected using yet another hidden variable that captures the latency between the ECG and the BP signals.

In Reference [52], a Convolutional Neural Network (CNN)-based approach is proposed for the detection and fusion of the annotations obtained from ECG and BP signals. Note that the CNN is able to extract the features by itself, and therefore, in contrast to the method from Reference [63], the inputs are the raw signals. Again, the CNN is trained with small intervals of data labelled as 1 or 0 depending on whether there is a heartbeat in the middle of the interval or not. Unlike the method from Reference [63], this proposal blends together detection and fusion.

## 4. Evaluation Metrics

To compare the different proposals of the literature, we will use a set of quantitative metrics. Those employed in the Physionet Challenge 2014 are a good starting point: gross sensitivity (Se), gross positive predictivity value (PPV), average sensitivity ($\bar{Se}$), average positive predictivity ($\bar{PPV}$), and overall score (Overall), which are computed as follows:

$$Se=\frac{100*TP}{TP+FN}$$

$$PPV=\frac{100*TP}{TP+FP}$$

$$\bar{Se}=\frac{100}{N}\sum_{n=1}^{N}\frac{TP_{n}}{TP_{n}+FN_{n}}$$

$$\bar{PPV}=\frac{100}{N}\sum_{n=1}^{N}\frac{TP_{n}}{TP_{n}+FP_{n}}$$

$$Overall=\frac{Se+PPV+\bar{Se}+\bar{PPV}}{4},$$

where N is the number of records, TP is the number of true positives (i.e., the number of heartbeats that are correctly detected), FP is the number of false positives (i.e., the number of detected heartbeats that do not appear in the ground truth), and FN is the number of missed heartbeats or false negatives (i.e., the number of heartbeats in the ground truth that are not actually detected). $TP_{n}$, $FP_{n}$, and $FN_{n}$ are the number of true positives, false positives, and false negatives for the record *n*, respectively. It must be noted that the main difference between the gross and average values relies on the different number of heartbeats per record. If all the records had the same number of heartbeats, then gross and average values would coincide. A tolerance interval of hundreds of milliseconds with respect to the ground-truth annotation of the database is typically given to a heartbeat to be considered as TP. For example, the Physionet Challenge 2014 considered a 150-ms tolerance interval.

In addition to the challenge metrics, in the literature, there are authors who use other metrics to evaluate the performance of their proposals. For example, the F1 score is defined as follows:

$$F1=\frac{100*2*TP}{2*TP+FN+FP}.$$

The average F1 can also be employed by averaging the F1 score obtained from the TP, FN, and FP values of each signal/record independently.

Additionally, the accuracy may also be employed for evaluation. This is defined as follows:

$$Accuracy=\frac{100*TP}{TP+FN+FP}.$$

All the evaluation metrics range between 0 and 100. For all metrics, the larger, the better.

## 5. Results and Discussion

Table 15 shows the results of the different techniques presented in this paper for the different databases from the evaluation metrics.

**Figure 3:**
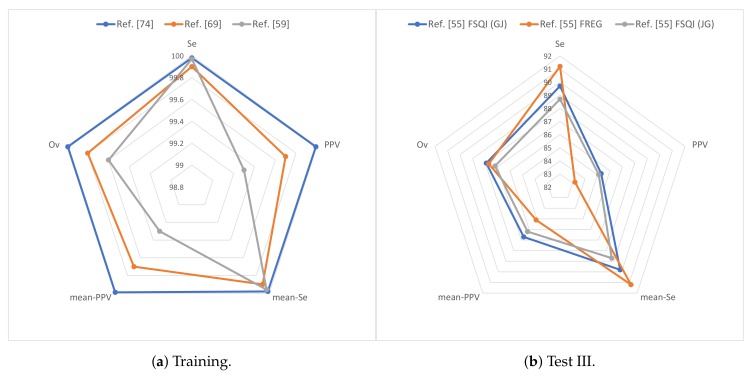
Results for the Physionet 2014 challenge database over (**a**) training and (**b**) test III data: $\bar{Se}$ is represented as mean-Se and $\bar{PPV}$ is represented as mean-PPV. “Ref.” stands for reference.

The Physionet 2014 challenge and its follow-up may be the most straightforward databases with in which comparison can be ascertained, since the largest amount of techniques are tested on those databases. Analysing their performance on the challenge database, the techniques obtained better performance on the training data than on the test data, as expected. The best overall performance on the training data (99.99% overall score) is obtained by the work presented in Reference [74], which employs ECG, BP, SV, EEG, and PPG signals; PSD computation as feature extraction; the GQRS algorithm for R-peak detection in ECG signal and a threshold-based approach in the rest of the signals, a majority voting approach for fusion; and Hjorth’s mobility and minimum heartbeat RR interval for missing annotations. However, this approach does not generalize well on test data (86.6%), probably due to overfitting. On the challenge test data (Phase III), the best overall performance (87.9%) is obtained by Reference [55]. It employs ECG, ABP, SV, and PPG signals and specific algorithms for heartbeat detection for each signal type. This approach generalizes better than the one presented in Reference [74] on unseen data. In addition, both the fusion approach based on the set of RR interval time series (FREG) and the one based on level agreement of two peak detectors (FSQI) may be more robust than the majority voting approach presented in Reference [74]. Figure 3 compares the three best results obtained over the Physionet 2014 challenge database, in which the overall metric has been chosen for evaluation ranking.

**Figure 4:**
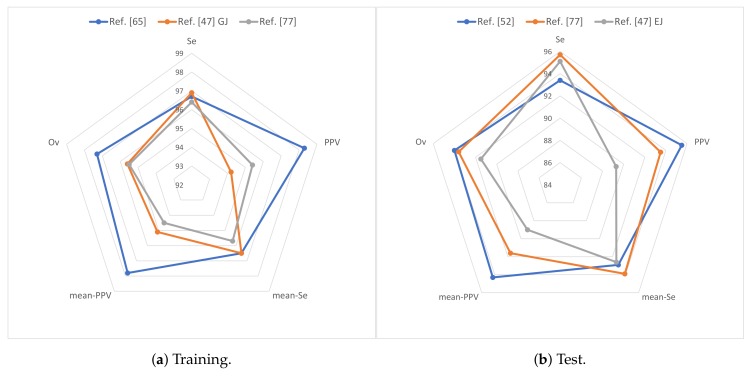
Results for the Physionet 2014 follow-up challenge database over (**a**) training and (**b**) test data: $\bar{Se}$ is represented as mean-Se and $\bar{PPV}$ is represented as mean-PPV.

Regarding the follow-up database, the technique presented in Reference [65] obtained the best overall performance on the training data (97.3%). This shows the usefulness of multi-lead ECG and BP signals, wavelet transform, correlation methods, and template matching approaches for robust heartbeat detection. On test data, the best overall performance is obtained the technique presented in Reference [52] (94.0%), which takes advantage of the ABP signal, CNNs, and a single detection and fusion stage for robust heartbeat detection. Figure 4 compares the three best results obtained over the Physionet 2014 follow-up challenge database, in which the overall metric has been chosen for evaluation ranking.

In the literature, there are authors who report better performances on the other databases than on the test challenge data (see Table 15). On the one hand, those databases already existed before the challenge was launched; therefore, more effort could have been devoted by researchers aiming to improve the performance of their algorithms over them. On the other hand, challenges are usually constructed with more difficult conditions (i.e., more noisy signals are involved and unseen test data is used for evaluation, to name a few), which makes the performance drop. According to the best results found for each individual database, EEG, EOG, and EMG signals (99.8% overall performance in the MIT-BIH Polysomnographic database [56]) and PPG signal (99.5% accuracy in the MIMIC database [53]) may outperform the performance of the most commonly used signal set (ECG and BP/ABP). However, some authors [70] note that the use of additional signals does not always improve the results, which may be due to the database used for evaluation and the algorithm/fusion approach employed. This could make the optimal signal set quite database-dependent.

Among those techniques that do use several signals (different from BP/ABP) to improve the detection, most of them require the signals to be labelled with their type (BP, EEG, etc.); only a few algorithms (see Reference [82] (ID)) are able to identify automatically the signal types.

Results have shown that signal preprocessing techniques such as band-pass filtering (with specific cutoff frequencies depending on the signal), notch filtering, and baseline wander suppression are also crucial for heartbeat detection. Wavelet transform and power spectral density have been shown to be suitable for feature extraction. Signal normalization and resampling are very commonly used during preprocessing. Techniques based on the variance of the delay values in the annotations, the U3 transform, the level agreement between two peak detectors, the SAI, correlation, and the deviation of the heartbeat from the rhythm of heartbeats have also shown their potential for measuring the quality of the signals to be used within the fusion.

Among the best individual heartbeat detection methods, GQRS, COQRS, JQRS, Open-Source Electrophysiological Toolbox (OSET), Slope Sum Function and Teager-Kaiser (operator) (SSF-TK), template matching, LDA, and Weighted Time Warping (WTW) algorithms are worth highlighting. Delay correction that aims to compensate the timestamp differences between the annotations output by the different signal detectors based on mean and median of the timestamps, the correlation method, or in a fixed delay value have been found to be useful.

Fusion techniques based on Hjorth’s mobility, RR intervals, delay values, majority voting, signal switching, detector level agreement, and CNNs have shown their potential for heartbeat detection. The review also shows that techniques which merge in a single-step detection and fusion are still a minority. This may be due to the fact that splitting detection and fusion eases the reuse of previous methods. However, the recent advances in machine learning enabling the development of end-to-end techniques may switch this tendency. In addition, deep learning has not yet been widely applied to the problem of ECG fusion. It should be noted that the technique that has obtained the best performance on challenge follow-up test database, that in Reference [52], together with that in Reference [63], are the only two techniques of Table 15 that use neural networks. This shows the potential of applying deep-learning techniques to heartbeat fusion.

## 6. Conclusions

An extensive review of the papers found in the literature that employ fusion of ECG with other physiological signals to enhance heartbeat detection has been presented. The best techniques are generally built from a few signals of interest (ECG, BP, and ABP) along with specific heartbeat detection algorithms depending on the signal type. Signal preprocessing based on different filters types, delay correction based on correlation methods, mean and median approaches, along with signal-quality assessment methods are often present in the approaches that have better performance. Fusion is accomplished by exploiting regularities and inconsistencies in the RR intervals obtained from the different signals; by selecting the most promising signal for the detection in every moment; by a voting process based on the detections over each individual signal; or by performing simultaneous detection and fusion using Bayesian techniques, hidden Markov models, or neural networks. Fusion techniques that aim to estimate the missing heartbeats have also been proven useful to enhance the performance.

We note that, to the best of our knowledge, there is a lack of proposals trying to use a new model that learns how to combine several fusion strategies (i.e., a *fusion of fusions*), in the same spirit of stacked generalizers in machine learning [124]. An issue with this approach (that may explain the lack of works) is that each individual fusion strategy must give *different* outputs so that they complement each other. We consider this an interesting line of work that is worth exploring. Another promising line may be the application of deep learning as a tool to detect beats over multiple signals and to fuse the results. The fact that the authors which have obtained the best performance on Physionet 2014 follow-up challenge test data have used a convolutional neural network [52] hints the potential of deep learning for heartbeat fusion.

Nowadays, it is difficult to compare research on multi-modal physiological signals for heartbeat detection due to the different databases and metrics employed in the different papers. Results obtained over the different databases cannot be compared, since these comprise different conditions: signal complexity, types of signals available, different noise levels, and different patient conditions, to name a few. Therefore, we encourage authors to pay attention to these issues during the evaluation. We highly recommend that future research should be evaluated using always the challenge follow-up data, since it is the most complete and challenging database and many proposals have already been evaluated over it. The MGH/MF Waveform database, which comprises a considerable amount of signal types and different patient conditions (stable and unstable patients) could be used as an additional database to test the techniques. Researchers may add other databases at their discretion, as long as they include the Physionet 2014 follow-up challenge database. Proposals must only employ training/development data for technique construction, so that the test data is only employed to validate the proposed technique. This permits a fair comparison between all the proposals for any database/metric. Moreover, the data used for system training and development should be specifically stated in the proposal, along with the system parameters, so that any author could replicate the results at their convenience.

Besides the databases, the metrics employed for evaluation also play a critical role when two or more techniques are compared. To that end, we recommend that all proposals report (at least) true positive, false negative, and false positive numbers per each record of the database. With these figures, all the metrics presented in Section 4 can be computed. The standardization in the databases and in the reported metrics would yield an optimal framework for comparison and would contribute to the progress of multi-modal fusion heartbeat detection.

## Figures and Tables

**Table 1:** Summary of the most common signals in the Physionet 2014 challenge database: “ECG” stands for electrocardiogram, “BP’ stands for blood pressure, “ART’ stands for arterial line, “PAP” stands for pulmonary arterial pressure, “RESP” stands for respiration, “EEG” stands for electroencephalogram, “N” stands for number of records, “$F_{s}$” stands for sampling frequency, and “min” stands for minutes. This table has been modified from Reference [22].

Data Set	ECG	BP	ART	PAP	RESP	EEG	N	$F_{s}$	Duration per Record
Training	100	100	0	0	0	0	100	250 Hz	≈10 min
Test Phase I	100	14	75	70	73	14	100	10–1000 Hz	≈10 min
Test Phase II	200	23	137	126	182	22	200	10–1000 Hz	≈10 min
Test Phase III	300	37	194	177	163	35	300	10–1000 Hz	≈10 min

**Table 2:** Number of training/test records of the Physionet 2014 follow-up challenge database: “EMG” stands for electromyogram and “EOG” stands for electrooculogram. This table has been taken from Reference [27] with minor modifications.

Signal	Training	Test
ABP	135	61
BP	25	116
Carbon dioxide level (CO_2_)	279	39
Central venous pressure (CVP)	123	57
ECG	210	200
EEG	25	110
EMG	8	44
EOG	8	44
PAP	122	6
General pressure (PRESS)	149	83
RESP	119	213
Oxygen level (SO_2_)	1	23
Stroke volume (SV)	1	23

**Table 3:** Summary of the MIMIC database. “N” stands for number of records, “h” stands for hours, “$F_{s}$” stands for sampling frequency, and “PLE” stands for fingertip plethysmograph.

Signal	N	Total Duration	$F_{s}$
ECG	100	24–48 h	500 Hz
ABP	100	24–48 h	125 Hz
RESP	100	24–48 h	125 Hz
Pulse oximeter	100	24–48 h	125 Hz
PAP	-	24–48 h	125 Hz
CVP	-	24–48 h	125 Hz
PLE	-	24–48 h	125 Hz

**Table 4:** Summary of the IMPROVE DL database: “N” stands for number of records, “h” stands for hours, “$F_{s}$” stands for sampling frequency, and “SAP” stands for systemic arterial pressure.

Signal	N	Duration per Record	$F_{s}$
ECG	66	24 h	100 Hz
SAP	66	24 h	50 Hz
PAP	59	24 h	50 Hz
CVP	66	24 h	50 Hz
${\mathrm{CO}}_{2}$	66	24 h	25 Hz
Airway oxygen	59	24 h	25 Hz
Airway flow	59	24 h	25 Hz
Airway pressure	66	24 h	25 Hz
EEG	7	24 h	100 Hz

**Table 5:** Summary of the MIT-BIH Polysomnographic database: “N” stands for number of records, “h” stands for hours, “$F_{s}$” stands for sampling frequency, “NTR” stands for nasal thermistor respiratory, and “IPR” stands for inductance plethysmography respiratory.

Signal	N	Duration per Record	$F_{s}$
ECG	18	2–7 h	250 Hz
BP	18	2–7 h	250 Hz
EEG	18	2–7 h	250 Hz
NTR	18	2–7 h	250 Hz
IPR	-	2–7 h	250 Hz
EOG	-	2–7 h	250 Hz
EMG	-	2–7 h	250 Hz
SV	-	2–7 h	250 Hz
Earlobe oximeter	-	2–7 h	250 Hz

**Table 6:** Reviewed papers that employ the different signals which can provide information for heartbeat detection: “BCG” stands for ballistocardiogram.

Group	Signal	Work
Directly related to cardiac activity	ECG	[42,43,44,45,46,47,48,49,50,51,52,53,54,55,56,57,58,59,60,61,62,63,64,65,66,67,68,69,70,71,72,73,74,75,76,77,78,79,80,81,82]
	ABP	[42,43,44,45,46,47,48,49,50,51,52,53,54,55,56,57,77,78,79,81,82]
	BP	[58,59,60,61,62,63,64,65,66,67,68,69,70,71,72,73,74,77,78,79,81,82]
	PPG	[53,54,55,74,81,82]
	SV	[55,66,72,74,79,81,82]
	PAP	[77,78,79,82]
	SAP	[75,76]
	CVP	[79,82]
	BCG	[80]
	PRESS	[81,82]
Indirectly influenced by cardiac activity	EEG	[56,57,67,68,73,74,82]
	EOG	[56,57,69,70,71,72,73,82]
	EMG	[56,57,71,73,82]

**Table 7:** Summary of the signal preprocessing techniques employed in the reviewed papers.

Filtering	Normalization	Downsampling/Resampling	Work
Low-pass	-	-	[44,45,57,73]
	-	YES	[71,80]
	YES	YES	[63]
Band-pass	-	-	[44,55,56,59,65]
	YES	-	[79]
	-	YES	[81]
High-pass	-	YES	[80]
	YES	YES	[63]
Notch	-	-	[65]
	-	YES	[80]
Smooth	-	-	[65]
Wavelet	-	-	[47,67,85]
	YES	-	[79]
Quadratic spline	YES	YES	[63]
Convolution	YES	-	[78]
Moving average	-	-	[66]
	YES	YES	[61]
Anti-aliasing	-	YES	[70]
Median	YES	YES	[52]
	-	-	[73]
Moving median	-	-	[57]
Mean	-	-	[57]
-	-	YES	[43]
-	YES	YES	[72]

**Table 8:** Summary of the feature-extraction methods employed in the reviewed papers: “LASTA” stands for lag-adaptive short-time autocorrelation function, “max” stands for maximum, “PAA” stands for piecewise aggregate approximation, “PSD” stands for power spectral density, “IMDF” stands for integer-multiplier digital filters, “min.” stands for minimum, “PR” stands for pressure ranges, and “AD” stands for average derivative.

Domain Type	Feature Extractor	Work
Time	U3 transform [86,87], first derivative	[70]
	LASTA [88], average magnitude difference [89,90], max. amplitude	[81]
	Symbolic discretization [91]	[58,72]
	PAA and signal discretization	[72]
	Slope-sum	[44,45]
	Derivative	[75]
Frequency	PSD	[74,82]
	FFT	[71]
	IMDF [92,93]	[62,77]
	Gaussian and moving average low-pass filters	[77]
Time–frequency	Wavelet	[47,49,63,65,67]
	Pulse score for scale determination [94]	[67]
	Frequency QRS power, kurtosis, max./min. amplitudes, PR, AD	[68]

**Table 9:** Summary of the Signal-Quality Assessment (SQA) methods employed in the reviewed papers: “SAI” stands for signal abnormality index, “PR” stands for pressure ranges, “AD” stands for average derivative, and “DWT” stands for discrete wavelet transform.

Type	SQA	Work
Statistical	Fisher’s g-statistic [96,97,98]	[59]
	Cross-correlation	[65]
RR-interval	R-peak intervals	[57]
	Peak distance, variance, number of annotations	[66]
	Heartbeat deviation	[56]
	Heartbeat comparison, dispersion	[79]
	Heart-rate variability [26]	[54]
	Number of annotations	[55]
SAI [95]	PR, AD	[42,46,47,50,55,68]
Detector-based	Multiple detector annotations	[43,44,45,47,50,55]
DWT	Second-level DWT coefficients	[64]
	Energy of the detailed DWT coefficients	[49]
Other	Band-pass filter, signal saturation, kurtosis	[44]
	RS slope detection	[73]
	Delay variance	[74]
	QRS complex power, kurtosis	[68]
	Hjorth’s mobility	[82]
	Sample entropy [99]	[48]
	U3 transform [86,87]	[55]

**Table 10:** Summary of the peak detection algorithms for ECG and BP/ABP used in the reviewed papers: “SSF-TK” stands for slope sum function and teager-kaiser (operator), “EPLTD” stands for Ep limited, and “OSET” stands for open-source electrophysiological toolbox.

Signal	Detector	Work
ECG	GQRS [34]	[42,55,59,60,64,66,72,73,79,82]
		[46,47,50,57,68,78,81]
	Pan-Tomkins [100,102]	[43,70,75,79]
	Gritzali [103,104]	[75,76]
	Hamilton-Tompkins [105,106]	[43]
	Christov [107]	[43]
	Afonso [108,109]	[43,48,49]
	Zong [23]	[43]
	COQRS [110]	[55]
	JQRS [111]	[47,55]
	Jinho [94]	[67]
	RS negative [112]	[57]
	Ecglib [113] (based on Reference [87])	[69]
	SSF-TK [114]	[56]
	Difference operator method [115]	[82]
	U3 detector [86,87]	[70]
	EPLTD [116] (based on References [100,105])	[47]
	OSET [117]	[65]
BP/ABP	WABP [23]	[42,45,46,47,50,56,60,64,66,72,78,79,81]
	RS positive (based on Reference [112])	[57]
	Li [118]	[48]

**Table 11:** Peak enhancing approaches used in the reviewed papers: “WTW” stands for weighted time warping, “CWT” stands for continuous wavelet transform, and “LDA” stands for linear discriminant analysis.

Type	Peak Enhancing	Work
Derivative-based	Equation (1)	[44,66,75,78]
	U3 transform	[69,70,86,87]
Template matching	WTW	[53]
	CWT+[117]+LDA	[65]
	FFT-based templates	[71]
	Full beat, clustered beat and statistical templates	[72]
Other	Envelope functions	[61,71,74,77,82]
	Wavelet-based enhancing	[63,65,79]
	Morphological filters	[62,77]
	Range filters	[61]
	Thresholding	[79]
	T-wave suppression filters	[80]
	Adaptive filters	[67]

**Table 12:** Summary of the peak detection methods used in the reviewed papers.

Type	Work
Local-maxima search	[54,57,66,78,82]
Bayesian	[81]
Machine learning	[52,63]
Data mining	[58]

**Table 13:** Summary of the delay correction approaches used in the reviewed papers.

Type	Delay Correction	Work
Constant for all data	Taken from literature	[52,64,78,79]
	Estimated from data	[45,75,81]
Patient-dependent but constant	Central tendency estimate	[47,48,49,55,56,57,66,73,74,82]
	Cross-correlation	[69,70]
Patient and time-dependent	Moving average filter	[60,61,67]
	Hampel filter [120,121]	[42]
	Windowed-correlation	[62,65,77]
	Physiological variance	[76]

**Table 14:** Summary of the fusion approaches employed in the reviewed papers: “SQI” stands for signal quality index, “HSMM” stands for hidden semi-Markov model, “DBN” stands for dynamic bayesian network, and “CNN” stands for convolutional neural network.

Type	Fusion	Work
RR-based	RR intervals	[42,46,57,58,59,61,64,72,73,78,79,81]
	Hampel filter [120,121]	[42]
	Hjorth’s mobility	[74]
	Nearest-neighbour	[43]
	Sandwich rule	[42,60]
	RR interval post-processing	[47,59,61,63,74,78,82]
Signal switching	Signal annotation selection	[47,48,49,55,57,62,65,66,68,77,79]
Voting	Majority voting	[67,74,79,82]
	Tukey weighted voting	[69,70]
	SQI, mean temporal location	[56]
	Mean correlation, template matching	[71]
	Bayesian inference	[54]
	Annotation score [122,123]	[76]
	AND/OR rules	[75]
Joint detection and fusion	Euclidean distance+DTW	[53]
	HSMM	[44,45]
	HMM+BN	[51]
	DBN	[50]
	CNN	[52]
Other	Manual	[80]

**Table 15:** Results obtained by the techniques presented in the reviewed papers: “Ov” stands for overall, “Ac” stands for accuracy, “rec” stands for records, “Ch” stands for challenge, “FU” stands for the follow-up of the challenge, “diff” stands for difficult, “CV” stands for cross-validation, “Ar” stands for arrhythmia, “Pol” stands for polysomnographic, and “NS” stands for noise stress. When a paper presents two or more techniques, these are denoted in the most left column with the corresponding ID referred across the paper: “GJ” for GQRS/JQRS, where GQRS was used as the baseline set of annotations and JQRS was used for signal-quality assessment evaluation; “JG” for JQRS/GQRS, where JQRS was used as the baseline set of annotations and GQRS was used for signal-quality assessment evaluation; “EG” for EPLTD/GQRS, where EPLTD was used as the baseline set of annotations and GQRS was used for signal-quality assessment evaluation; “EJ” for EPLTD/JQRS, where EPLTD was used as the baseline set of annotations and JQRS was used for signal-quality assessment evaluation; “G+W” for GQRS+WABP, where detections from both algorithms are fused; and “G+O” for GQRS+OWN, where detections of both algorithms are fused. The work marked with “*” presents the results on the database composed of training and test data. All results are given in %. For results higher than 99.9, the second decimal is shown so that the best performance can be seen. Results from Reference [69] have been presented as in the original paper.

Work	Database	Se	PPV	$\bar{\mathrm{Se}}$	$\bar{\mathrm{PPV}}$	F1	$\bar{F1}$	Ov	Ac
[58]	30 rec Ch training	65.3	72.1	51.7	67.2	-	-	-	-
[59]	Ch training	99.97	99.3	99.96	99.3	-	-	99.6	-
	Ch test III	83.3	79.8	83.8	77.8	-	-	81.2	-
[42]	FU test	91.1	87.1	89.4	87.2	-	-	88.7	-
	100 diff rec FU training	93.2	88.5	92.1	89.9	-	-	90.9	-
	CV 100 diff rec FU training	92.5	88.6	91.3	90.0	-	-	90.6	-
[66]	MGH/MF training	-	-	96.9	96.8	-	-	-	-
	Ch training	-	-	-	-	-	-	99.96	-
	Ch test I	-	-	-	-	-	-	90.0	-
	Ch test II	-	-	-	-	-	-	83.8	-
	Ch test III	-	-	-	-	-	-	84.3	-
[73]	Ch test III	-	-	-	-	-	-	86.4	-
[74]	Ch training	99.98	99.99	99.98	99.99	-	-	99.99	-
	Ch test II	85.2	86.7	85.7	87.3	-	-	86.2	-
	Ch test III	88.9	83.8	88.5	85.3	-	-	86.6	-
[69]	Ch training	>99.9	99.7	>99.9	99.7	-	-	99.8	-
	Ch test I	86.6	95.7	85.5	88.0	-	-	88.9	-
	Ch test II	73.4	80.5	75.3	75.6	-	-	76.3	-
	Ch test III	84.6	86.8	82.9	83.5	-	-	84.2	-
[55] FSQI (GJ)	MGH/MF training	94.3	96.4	93.9	95.9	95.4	-	95.2	-
	Ch test III	89.7	85.3	89.8	86.7	87.5	-	87.9	-
[55] FSQI (JG)	MGH/MF training	94.0	96.3	93.7	96.2	95.2	-	95.1	-
	Ch test III	88.7	85.1	88.7	86.2	86.8	-	87.2	-
[55] FREG	MGH/MF training	96.2	95.6	95.8	95.6	95.9	-	95.8	-
	Ch test III	91.2	83.2	91.2	85.1	87.0	-	87.7	-
[44]	Ch test III	-	-	-	-	-	-	83.5	-
	5150 rec MGH/MF	-	-	-	-	-	-	92.7	-
[71]	47 rec MIT-BIH Ar	99.8	99.0	-	-	-	-	-	-
	Ch training	99.9	99.96	-	-	-	-	-	-
	Ch test III	83.6	84.8	-	-	-	-	83.7	-
[60]	Ch training	99.9	-	99.96	-	-	-	-	-
	Ch test III	87.8	-	85.2	-	-	-	86.7	-
	Ch training	-	-	-	-	-	-	99.6	-
[80]	4 healthy subjects	-	-	-	-	-	-	-	88.0
[53]	MIMIC	-	-	-	-	-	-	-	99.5
[67]	Ch training	-	-	-	-	-	-	99.6	-
[64]	200 rec Ch	96.4	94.5	95.9	94.9	-	-	95.4	-
[61]	Ch training	99.9	-	99.93	-	-	-	-	-
	MIT-BIH Ar training+test	98.6	-	99.7	-	-	-	-	-
	MIT-BIH NS test	94.9	-	92.0	-	-	-	-	-
	European ST-T	99.91	-	99.9	-	-	-	-	-
	MGH/MF training+test	98.7	-	98.3	-	-	-	-	-
	MIT-BIH Pol	99.9	-	99.7	-	-	-	-	-
[62]	Ch test I	-	-	-	-	-	-	89.2	-
	Ch test II	-	-	-	-	-	-	85.9	-
	Ch test III	-	-	-	-	-	-	85.1	-
[63] NN	Ch training	99.94	99.96	-	-	-	-	-	-
	Ch test III	91.6	87.9	-	-	-	-	-	-
	FU training	95.5	92.2	-	-	-	-	-	-
[63] BT	Ch training	99.95	99.96	-	-	-	-	-	-
	FU training	96.5	92.3	-	-	-	-	-	-
[54]	MIMIC	-	-	-	-	-	-	-	77.0
[72]	100 rec Ch test	-	-	-	-	-	-	88.0	-
[56]	FU training	95.3	95.0	94.8	94.6	95.2	94.7	94.9	-
	FU test	92.7	90.4	91.6	88.9	91.6	90.2	90.9	-
	MIT-BIH Pol	99.9	99.6	99.9	99.7	99.8	99.8	99.8	-
[79]	FU *	-	-	95.5	96.0	95.6	-	-	93.1
[82] PSD	FU training	94.6	92.4	-	-	-	-	93.5	-
	FU test	89.0	87.5	86.4	85.3	-	-	87.1	-
	100 rec MGH/MF	90.8	96.7	-	-	-	-	93.7	-
[82] ID	FU training	95.4	93.3	-	-	-	-	94.3	-
	FU test	90.7	90.2	89.6	89.6	-	-	90.0	-
	100 rec MGH/MF	97.1	97.8	-	-	-	-	97.4	-
[43]	MGH/MF training+test	90.6	96.7	-	-	-	-	93.7	-
[70]	FU training	95.9	91.4	95.7	92.3	-	-	93.8	-
	FU test	92.7	87.4	91.1	87.0	-	-	89.5	-
	MIT-BIH Pol	99.98	99.0	-	-	-	-	-	-
[45]	FU training	94.5	96.5	94.8	95.6	-	-	95.3	-
	FU test	92.8	88.5	89.7	85.4	-	-	89.1	-
[81]	Ch training/test	95.7	95.5	96.1	96.3	-	-	95.9	-
	FU training/test	91.0	91.9	89.4	90.5	-	-	90.7	-
	MIT-BIH NS	86.3	80.3	86.1	80.4	-	-	83.3	-
[46]	Ch training	99.9	99.96	-	-	-	-	-	-
	Ch test III	82.1	84.1	-	-	-	-	-	-
[76]	IMPROVE	-	-	-	-	-	-	-	63.6
[57]	Ch test III	87.0	85.8	87.6	85.2	-	-	86.4	-
	FU test	88.6	88.3	88.0	87.5	-	-	88.1	-
[77]	LTST	96.5	94.1	96.0	94.0	-	-	95.1	-
	MGH/MF training+test	-	-	95.2	93.2	-	-	-	-
	Ch training and FU training	98.1	97.5	97.8	97.2	-	-	97.7	-
	FU training	96.4	95.4	95.7	94.5	-	-	95.5	-
	FU test	95.7	93.5	93.9	91.6	-	-	93.6	-
[51]	FU training	-	-	94.0	93.0	-	-	-	-
[49]	Ch training	99.7	99.92	99.7	99.91	-	-	-	-
[47] GJ	FU training	96.9	94.2	96.5	95.1	95.5	-	95.6	-
	FU test	94.0	88.8	91.6	88.8	91.3	-	90.8	-
[47] EG	FU training	96.8	93.7	96.5	94.9	95.2	-	95.4	-
	FU test	94.0	87.7	91.6	88.4	90.8	-	90.4	-
[47] EJ	FU training	94.4	93.5	93.8	94.4	94.0	-	94.0	-
	FU test	95.1	89.3	92.6	89.0	92.1	-	91.5	-
[68]	Ch training	-	-	-	-	-	-	99.97	-
	Ch test III	-	-	-	-	-	-	86.3	-
[78] G+W	FU training	92.1	91.6	92.2	93.0	91.8	92.5	92.2	-
	FU test	89.8	85.8	87.3	86.3	-	-	87.3	-
[78] OWN	FU training	90.9	89.5	91.0	90.8	90.2	90.7	90.6	-
	FU test	87.8	85.1	85.3	83.3	-	-	85.4	-
	MIT-BIH Ar training+test	-	-	99.1	99.8	-	-	-	-
[78] G+O	FU training	94.5	94.0	94.4	94.4	94.2	94.2	94.3	-
	FU test	91.2	88.1	88.9	87.3	-	-	88.9	-
[48]	Ch training	99.7	99.91	99.7	99.91	-	-	-	-
[65]	FU training	96.7	98.3	96.5	97.8	-	-	97.3	-
	MGH/MF training+test	96.3	97.0	96.2	96.9	-	-	96.6	-
[50]	Ch training	99.6	99.9	99.6	99.9	-	-	-	-
	FU training	94.1	93.6	93.1	93.2	-	-	-	-
	MIT-BIH Pol	99.7	99.7	99.6	99.7	-	-	-	-
	MGH/MF training+test	95.4	96.1	95.3	95.3	-	-	-	-
[75] (and)	IMPROVE	-	-	-	-	-	-	-	86.1
[75] (or)	IMPROVE	-	-	-	-	-	-	-	92.1
[52]	FU test	93.4	95.5	92.9	94.3	-	-	94.0	-
	23 rec MIT-BIH Ar	99.93	99.91	99.94	99.91	-	-	99.92	-
